# The Experiences of Nurses and Physicians Caring for COVID-19 Patients: Findings from an Exploratory Phenomenological Study in a High Case-Load Country

**DOI:** 10.3390/ijerph18179002

**Published:** 2021-08-26

**Authors:** Moawiah Khatatbeh, Fadwa Alhalaiqa, Aws Khasawneh, Ala’a B. Al-Tammemi, Haitham Khatatbeh, Sameera Alhassoun, Omar Al Omari

**Affiliations:** 1 Department of Basic Medical Sciences, Faculty of Medicine, Yarmouk University, Irbid 21163, Jordan; 2 Faculty of Nursing, Philadelphia University, Amman 19392, Jordan; fhalaiqa@philadelphia.edu.jo; 3 Department of Neurosciences, Jordan University of Science and Technology, King Abdullah University Hospital, Irbid 22110, Jordan; agkhasawneh3@just.edu.jo (A.K.); sjalhassoun@just.edu.jo (S.A.); 4 Department of Family and Occupational Medicine, Faculty of Medicine, University of Debrecen, 4032 Debrecen, Hungary; alaa.tammemi@med.unideb.hu; 5 Doctoral School of Health Sciences, University of Debrecen, 4032 Debrecen, Hungary; 6 Doctoral School of Health Sciences, Faculty of Health Sciences, University of Pécs, 7621 Pécs, Hungary; Khatatbeh.haitham@etk.pte.hu; 7 College of Nursing, Sultan Qaboos University, Al-Khoudh, Muscat 123, Oman; o.alomari@squ.edu.om

**Keywords:** COVID-19, HCWs, health professionals, Jordan, lived experience, pandemic, phenomenology, support

## Abstract

Various changes have affected health services delivery in response to the repercussions of the COVID-19 pandemic, and this may exhibit unprecedented effects on healthcare workers (HCWs). This study aimed to explore the lived experience of physicians and nurses caring for patients with COVID-19 in Jordan. An interpretative phenomenology approach was used, and sampling was purposively performed. Data were collected through semi-structured interviews using an online meeting platform (Zoom^®^). Interviews were audio-recorded, transcribed verbatim, and analyzed. The data were obtained from 26 physicians and nurses caring for patients with COVID-19. The mean age of the participants was 29.41 years (SD = 2.72). Six main themes and 17 subthemes were identified: (i) emotional reactions; (ii) preparation; (iii) source of support; (iv) extreme workload; (v) occupational challenges, and (vi) work-related concerns. The results showed that nurses and physicians caring for COVID-19 patients in Jordan were experiencing mental and emotional distress and were practicing under inadequate work conditions. This distress could be multifactorial with personal, organizational, or cultural origins. Our findings may guide policymakers to consider the potential factors that significantly affect working environment in healthcare settings, the physical and mental wellbeing of HCWs, and the required professional training that can help in enhancing resilience and coping strategies amidst crises.

## 1. Introduction

Coronavirus disease 2019 (COVID-19) is rapidly spreading internationally. As of August 2021, about 202 million cases were confirmed worldwide, with more than 4.2 million deaths [1]. Healthcare workers (HCWs) are vital resources for every health care system. Their health and safety are crucial not only for continuous and safe patient care but also for control of any outbreak [2]. The rapidly evolving pandemic has impacted the entire global healthcare system with an increased demand for health care services. However, the increasing demand led to reallocating healthcare providers who lack sufficient experience in dealing with such emerging diseases to serve in the frontlines to counter the disease, thus putting them at a higher risk for contracting the infection due to the high infectivity rate of the COVID-19 virus. Despite the recent approval of various vaccines against COVID-19, and the start of vaccine rollouts in many countries globally, the pandemic is still striking many countries very severely considering the lack of sufficient vaccine supplies, the evolving viral variants, and the effect of pandemic fatigue [3,4,5]. Additionally, the pandemic crisis has impacted various life domains and sectors, thus afflicting day-to-day activities and personal practices [6,7].

Traumatic events can cause a wide range of psychosocial and physical impacts on individuals [8]. HCWs who gave care for patients during the severe acute respiratory syndrome (SARS) and the Middle East Respiratory Syndrome (MERS) outbreaks were subjected to many health hazards. They served under extraordinary stress due to the high risk of infection, stigmatization, understaffing, and uncertainty [9,10]. Quantitative studies have shown that frontline healthcare providers who treat patients with COVID-19 have greater risks of mental health problems, such as anxiety, depression, insomnia, and stress [11]. Frontline physicians and nurses who had no expertise in dealing with infectious diseases had additional challenges when they shifted into an entirely new work environment [11,12]. The widespread transmission of the virus has intensified challenges in healthcare systems in most countries. The HCWs became confused within the treatment system due to the unpredictable nature of the disease, social isolation, and the increasing infectivity and fatality rates [13]. Working under stress, the daily influx of patients to hospitals, low hospital capacity, and the lack of medical facilities and staff have also made offering care awkward [14].

In addition to the stressful conditions, HCWs are exposed to serious risks and even death while administering care for patients with COVID-19. This led to an increased mental and emotional distress. In Iran, a qualitative study showed that nurses working in the wards and care centers designated for patients with COVID-19 are experiencing mental and emotional distress [15]. Similarly, nurses caring for patients diagnosed with COVID-19 in Turkey were adversely affected, both psychologically and socially, by the pandemic; they used short-term coping strategies, and they needed psychosocial support. They also faced stigmatizing attitudes and were at risk for secondary traumas due to witnessing disease and death [16]. In China, healthcare providers were interviewed and reported being challenged by working in a new context, exhaustion due to heavy workload, fear of becoming infected and infecting others, and feeling powerless to handle patients [17]. However, the detailed life experience of the HCWs in the COVID-19 wards and their well-being during this time is scanty.

Jordan, a country located in the Eastern Mediterranean Region (EMR), effectively succeeded in preventing the spread of COVID-19 during the first six months after confirming the first case in early March 2020 [18,19]. However, the next months witnessed a massive number of COVID-19 cases and deaths which resulted in a significant burden on the country’s healthcare system [4,20]. Consequently, this led to the overwhelming of both the healthcare system and the health workforce. Besides, the pandemic existence in the country has imposed significant psychosocial and health impacts on HCWs and various components of the society as well [4,12,21,22,23,24,25,26]. In addition, various changes have affected health services delivery in response to the repercussions of the pandemic and its mitigation measures in Jordan, and this may exhibit unprecedented effects on the HCWs [12,27,28]. This study aimed at exploring the lived experiences of HCWs in Jordan during the COVID-19 pandemic to gain insights into the most efficient ways to support them. Our study is expected to provide policymakers and decision-makers in Jordan and the EMR with in-depth insights into the personal, institutional, and organizational factors that may impact the HCWs while working amidst the COVID-19 crisis. The findings of our study will be helpful for many countries, especially low and middle-income ones.

## 2. Materials and Methods

### 2.1. Study Design

An exploratory qualitative study was conducted among physicians and nurses caring for patients with COVID-19 in Jordan. In specific, an interpretative phenomenological analysis (IPA) design was utilized to explore the lived experiences of the participants. IPA is useful when there is a paucity of information available regarding the phenomenon under study and when the researchers want to generate a rich description of this phenomenon [29,30].

### 2.2. Study Setting, Participants and Sampling

Participants were recruited through purposive sampling. Participants who were (i) Jordanians; (ii) registered nurses or/physicians; and (iii) in direct contact with COVID-19 patients for the last three months, were included in the current study. The study setting was two hospitals that were the main designated treatment and care centers for patients with COVID-19 in the country, and where most COVID-19 confirmed cases that need admission as well as tertiary care are referred to. The first facility (hospital 1) is one of the public-based tertiary care centers that was established in Amman, the capital city of Jordan, with occupancy of 441 beds (around 3.0% of the total number of hospital beds in Jordan for the year 2017), while the second facility (hospital 2) is one of the tertiary care centers that was established in Irbid city in the north of Jordan, and It has 542 beds (around 3.7% of the total number of hospital beds in Jordan for the year 2017). According to the latest available data from the World Health Organization’s Global Health Workforce Statistics and the World Bank, Jordan had 2.82 nurses and midwives per 1000 people in 2018, while around 2.32 physicians per 1000 people in 2017 [31,32] Sampling continued until data saturation was reached and no new themes emerged.

### 2.3. Data Collection

The researchers conducted 26 interviews with 26 Participants. A copy of the ethical approval was sent to the department heads of the targeted hospitals to interview nurses and physicians. Flyers were posted on the units’ notice boards. The flyers included the purpose of the study, inclusion criteria, and the researchers’ contact details. Participants who were interested in the study were able to communicate with the researchers either by phone or email, any further concerns were answered, and the interested participants were invited to take part in the study. Participants who agreed to take part in the study were invited to attend an online interview at an agreed time. The interviews were conducted online using a Voice over Internet Protocol (VoIP) application called Zoom^®^, because of the strict isolation procedures, which prohibited the entry of miscellaneous people to the COVID-19 units and to protect the interviewers from any risk associated with meeting high-risk individuals.

### 2.4. Interviews with HCWs

A semi-structured individual interview was used to collect data. In-depth interviews were conducted with participants and recorded. Each interview lasted between 45–60 min. At the beginning of each interview, the researchers collected verbal consent from all participants before recording and asking guided open-ended questions to establish a rapport. Then, a broad data-generating question was first used: “Please tell me about your experience of taking care of patients with COVID-19”, and open-ended follow-up questions were used to obtain detailed descriptions. Various probing techniques were utilized to enable physicians and nurses to explicitly express their experiences and enhance the depth of the discussions. The interviewer ended the interview by asking participants whether they want to add any story. Examples of these questions are shown in Table 1.

The interviews were performed by three researchers (A.B.A.-T., H.K., S.A.) who had prior experience in qualitative research. To enhance the validity, training was conducted to all researchers about how to conduct the interviews in the same manner. The interviews were conducted at convenient times to both the participants and the researchers between January and April 2021. The interviews were recorded after getting consent from all participants. Participants’ age, marital status, and years of work experience were obtained at the beginning of the interview.

### 2.5. Data Analysis

The meetings were recorded and transcribed verbatim to Microsoft Office Word^®^ by the research team. Then, the transcripts were double-checked against the recorded interview by different research team members to ensure their accuracy using the Arabic language. Two independent researchers (M.K., F.A.) analyzed the data using the following steps as guided by Smith, Flowers and Larkin (2009) [38]:

(i) Read and reread the transcript several times to allow familiarity with the data and get a better understanding; (ii) During the reading rounds, significant phrases, words, and insights in the transcripts were annotated and highlighted; (iii) Notes were prepared carefully and documented; then, researchers searched for connections between the notes and clustered them in groups to allow identification of the emerging subthemes; (iv) Researchers searched for connections between the emerging subthemes using strategies such as abstraction, subsumption, and polarization; (v) Same steps were repeated with each interview transcript; (vi) The last step involved reviewing the subthemes from all interviews to establish connections between them using abstraction, subsumption, and polarization to cluster the subthemes into major themes. Once the process was completed, the two researchers met and discussed differences in the analysis until they reached a consensus about the themes, subthemes, and exemplars used to describe the theme or subtheme. Inter-coder reliability was approximately 90%, and the whole research team met and reviewed the overall analysis process to ensure its clarity.

### 2.6. Trustworthiness

Several strategies were used to ensure credibility and trustworthiness. Credibility was achieved by in-depth interviews followed by peer debriefing. Two coauthors analyzed the transcripts independently. Findings were then compared and discussed by the team until reaching consensus on themes, theme clusters, and categories were achieved. The researchers used also the “audit trails” approach by detailing the methodology and analysis sections. A team member, who is an expert in qualitative research, was asked to audit the methods and data analysis before giving positive feedback about the clarity of the methods and data analysis sections. The last technique used was “grounding in examples” to allow readers to make conclusions about the quality of the analysis. The themes were presented with actual participants’ statements from the transcripts to support each theme and subtheme.

Transferability was established by considering variations of participant characteristics and sufficient quotations collected through in-depth interviews. The audit trail was maintained to ensure all analysis steps could be traced back to original interviews. To maintain the trustworthiness and credibility of the findings and avoid inaccurate translation issues, translations into English were performed after generating themes [39].

### 2.7. Ethical Considerations

The present study was approved by the research ethics committee at Yarmouk University, Jordan. The study objectives and voluntary nature of the study were explained to participants. Written and verbal informed consents were obtained from all participants before recording and conducting the interviews. Anonymity was maintained as the researchers did not collect any personal information from the participants. All recorded interviews and transcripts were saved on a password-protected computer in the office of the principal investigator. Throughout this study, we followed the standards for reporting qualitative research in accordance with the Consolidated Criteria for Reporting Qualitative research Checklist (COREQ) (Supplementary File S1). All study procedures were carried out following the ethical standards of the Declaration of Helsinki.

## 3. Results

### 3.1. Study Participants

Our sample consisted of 26 participants (10 physicians and 16 nurses). All participants took care of patients with COVID-19 in the hospitals at which they were employed. Physicians were deployed to COVID-19 wards from medical and emergency wards (ER), while nurses were deployed to COVID-19 wards from various wards including medical, surgical, ER, and outpatient clinics. Participants differed by socio-demographic characteristics as illustrated in Table 2. Thematic redundancy was achieved by the 22nd interview, and four participants were then interviewed to confirm this redundancy.

### 3.2. Participants Characteristics

The mean age of participants was 29.9 years, and the average experience duration was 6.5 years (average experience as a health professional). Table 2 shows the demographic characteristics of the participants.

### 3.3. Themes and Sub-Themes Categorization

Six themes emerged in the current study from interviewing 26 HCWs working with COVID-19 patients; (i) emotional reactions; (ii) preparation; (iii) source of support (iv) extreme workload; (v) occupational challenges and (vi) work-related concerns. The themes and sub-themes are summarized in Table 3.

### 3.4. Theme One: Emotional Reactions

Dealing with COVID-19 patients for the first time was associated with many feelings among physicians and nurses. These feelings included fear, tension, anxiety, and worries resulted in increased stress levels.

• *The feeling of fears, worries, and anxiety*.

Most of the participants expressed a fear feeling of being infected or spreading the infection to their family.

“*It was a mixed feeling of fear and anxiety, especially the worry about consequences on my wife and children… I experienced huge psychological stress and anxiety from the idea that I might be a carrier of the disease (COVID-19). My fear developed into an obsession, especially after I have collected several positive samples*” (male physician). This feeling extended to be a fear of being a source of infection to other patients; this was clearly reported by a male nurse who was working in the operation room: “*I felt anxious and fearful from the possibility of transmitting the infection to additional patients through the operation room*”.

Meanwhile, few participants faced the situation and tried to minimize its effects by accepting it, telling themselves that it is their duty. A female physician who works in the hospital said, *“In fact, I did not exhibit any worries. At the beginning, I dealt with COVID-19 patients just like any normal working day, supported by the idea; it is my duty, and I must bear it*”. With time, some participants started to cope with the new work conditions. A female nurse stated: “*This feeling (fear and stress) began to fade with time, as I became more familiar with the disease, and I became competent in wearing protective gears and other PPEs*”.

• *Factors increased the fear, stress, and anxiety.*

Most participants used rationalization as a defense mechanism by stating the cause of their feeling of fear, stress, and anxiety in different ways. They verbalized having an old age parent with co-morbidity as the cause of their fear: A female nurse said*, “I worried about spreading the infection to my family, they are old and have chronic diseases. If they got the infection, it might threaten their lives… in fact, this point was the main source of my stress*”.

Some participants expressed that lack of knowledge about COVID-19 increased their anxiety and stress. A male physician who works in the hospital said, *“The situation was a bit confusing and stressful, mainly because there was not enough information about the disease… every day we hear something new about the disease (COVID-19)… mode of transmission and treatments*”. Although some participants expressed inadequate knowledge and unclear situation, they tried to calm, comfort, and reassure their patients. A female physician in the hospital said*, “In the beginning, everything was not clear to me, so I had a sense of fear about my health, but despite that, I was trying to show the patient a feeling of comfort and reassurance so that he could receive treatment easily… we all know that psychological status of the patient can affect their immunity level*”.

### 3.5. Theme Two: Preparation

Health care providers in the current study articulated two types of preparation that enhanced their resilience: organizational and personal.

• *Organizational preparation.*

Most participants stated that the organization provided adequate personal protection equipment (PPEs) which played a role in reducing stress. A female physician in the hospital said, *“the hospital provided all means to reduce infection and motivate us; this included all medical information (such as an introduction to the disease, its symptoms, and methods of prevention), and all preventive equipment was available to HCWs (masks, gloves, face protectors and work clothes)*”. A male nurse said*, “Availability of PPEs, masks, gloves… all these gave us a feeling of support and increased our resilience… reduced our stress*”.

The response of the organization by meeting the needs of HCWs was also an important factor that enhanced resilience: “*In the beginning, the hospital was not fully prepared, but after the repetitive claims, all protective equipment was provided… this was a great response. I felt more comfortable*”. A female physician.

Although the availability of PPEs was a positive factor to support resilience, some participants were challenged with using PPEs (limitation of movement, feeling of hotness and sweating, marks at face and body after using PPEs for long hours). A female nurse said, “*The PPEs did not allow me to move freely… had difficulty in using it… it made me feel suffocated*”. Providing appropriate response and adequate care for colleagues who got infected with the virus was another factor that helped in alleviating participants’ stress and made them feel reassured and comfortable. A male physician reported, *“when one of the healthcare providers got ill, I felt reassured due to the high level of care they received*”.

• *Personal preparation.*

Healthcare providers in the current study tried to enhance their resilience by following safety precautions at work and everywhere else. A female physician said, *“I was concerned about infection even while wearing PPEs. So, I was committed to applying all precautionary measures of wiping and proper washing of hands*”. Additionally, some of our participants followed a healthy lifestyle (e.g., appropriate food, practicing exercise, positive way of thinking). A male physician said, *“I tried to be positive in everything, I started listening to the Quran to replenish my inner feeling, I started to follow an exercise program, eat a lot of fruit and vegetables, drink a good amount of water…*”. On the other side, some participants isolated themselves from their families. A male physician said, *“For four months, I have sent my wife and kids to her family house, I’m living alone. Every week, I do the PCR test and when the result is negative, I go out using a face mask, gloves, and maintaining social distancing… being a physician means to sacrifice… and our homeland deserves this giving*”.

### 3.6. Theme Three: Source of Support

There were many sources of support to our participants who worked with COVID-19 patients (e.g., family, friends, organizational, and community support).

• *Family and friend support.*

Many HCWs who engaged in the current study expressed that their family provided encouragement and support to them through a variety of methods (e.g., words of thanks, frequent contact). A male nurse said*, “We were encouraged and got support from family, friends, and patients… their words of thanks were very motivating to us “.* Besides, a female physician said, *“The family has been very supportive to us (psychologically); perhaps we have not been in constant contact with them, but every day we have some time, even if it is short, to contact children, parents, brothers, and friends. This contact increased satisfaction at work and motivated us to give more to protect them*”. However, few participants reported that they did not need any support as the COVID-19 pandemic is a special situation and professionally they must be responsible, and it is their duty. A female nurse stated*, “I did not get any support… I did not even need any… I work with this situation because I have to sacrifice for my country… this is my professional responsibility… working with COVID19 patients was my choice… they asked us if we want to work or not*”.

• *Organizational and colleagues’ support.*

Physicians and nurses in the current study viewed support from colleagues as another source of support that encouraged them to work. A male physician said, “*As colleagues, we were very cooperative; when I felt tired during work, my colleague did my job and asked me to get some refreshing air*”. Participants have also perceived their organizations as an important source of support when they provided HCWs with all needed equipment. A female physician said, “*I received support from everyone in the hospital and community associations… all required equipment and requirements were available to all staff… and everyone was happy to help… the support was not only material, they carried out some activities and honors to raise our morale… the work division and organization was one of the most important things that helped the crew to feel comfortable, which led to increasing our giving*”.

• *Other sources of support (community and self).*

Most participants expressed that the greatest support was coming from His Majesty the King of Jordan, Abdullah II bin Al-Hussein, who gave the HCWs a push to work hard.

“*The greatest psychological support came from the words of His Majesty the King, who is the leader and inspirer in the field. His Majesty was leading the battle against this virus*” (male physician). Although our participants articulated different sources for support, some of them complained of lacking financial support. A male nurse said, “*I think we needed financial support because we did not get any day off, but, unfortunately, we did not get any*”. A male physician reported, “*I think there was a kind of important support that we needed… but we did not get it… it was financial support. There was a nurse who worked with us for 17 days… she has kids… I think that financial support can compensate for hard work, decrease most stress, and may lead to job satisfaction*”.

### 3.7. Theme Four: Extreme Workload

In the current study, health care providers stated that long working hours with COIVID-19 patients had affected various aspects of their life (e.g., social, physical, psychological).

• *Social impact.*

Long working hours have harmed the participants’ social life by reducing their socialization. A male physician said, “*Of course, long working hours had a great influence on our social relations, whether at home or with family, by virtue of the fact that it was possible to be a carrier of the disease without having symptoms. For example, I could not visit my mother even though she was living a few meters away from my home… it was a bad feeling and, undoubtedly, it greatly affected our focus*”.

• *Physical impact.*

Many physical changes were reported by health care workers because they worked for long hours and being mentally occupied. A male physician reported, “*I worked for 16 h daily, without getting enough sleep, … because of the high tension, continuous thinking and lack of sleep, I started having hair loss*”. Some participants complained of having headaches and muscle pain, so they started using analgesics. A female physician said, *“As a result of long working hours, the helmet caused a terrible headache, so I used Panadol Extra 4–6 pills per day, and sometimes I used muscle relaxants to reduce pain in the lower extremities*”. Some health care providers behaved in a maladaptive way. They started using sleeping pills to overcome their insomnia. A female nurse said, “*Throughout my work in the isolation department, I suffered from insomnia, and I was unable to sleep… the maximum number of hours I slept was 3 h using medications to help, such as anti-histamines… Most of my sleep attempts were unsuccessful because I woke up terrified with nightmares, I do not remember anything of them, but they are all related to the virus*”. Other participants reported changes in their eating habits which resulted from their fear and stress. A male physician said, “*I felt horror to the extent that I did not eat as usual and did not feel hungry either (loss of appetite), despite the small amount of food that I ate*”.

• *Psychological impact.*

Our participants stated that long working hours adversely affected their psychological status; they had anxiety, tension, fear, anger feeling, and nervousness. A male physician said, “*Working in the isolation department was psychologically harmful to me, I had great anxiety, tension, and fear of entering the rooms of COVID-19 patients (fear of being infected)… I had difficulty working under this kind of psychological pressure… and because of the aversion of dealing with patients, some of them looked at me with resentment*”. A female nurse from the isolation unit reported, “*I felt their (patients’) fear, depression, and some of them verbalized having depression. They were depressed even though their health was excellent. I imagined myself in their place and how I would need support and communication with the surroundings. The idea of isolation is very annoying, even if they can communicate on the phone, but that does not substitute for direct contact with their loved people*”. Additionally, the patient’s psychological and physical status affected the psychological wellbeing of the health care workers. A male physician said, “*some cases stayed more than 50 days in the hospital…, this greatly affected my psychological status… those patients had a state of depression mainly after each time when they got a positive PCR result… Another patient was a young boy who was crying and had a panic-like state…, the whole team felt sad and was sympathetic with him*”.

• *Cognitive impact.*

Dealing with COVID-19 patients for long hours had a negative impact on the way of thinking of HCWs; it affected their concentration and thoughts. In terms of concentration, some of our participants reported their focus to be directed to the virus and its associated infection (a tunnel through and vision), even their ability to focus on other jobs than the virus was reduced. A male physician said, “*All my thoughts were centered on the infection with the virus, transmission of infection and the occurrence of complications… for me, the rest period was a source of great psychological fatigue because I was left with my fears and anxieties, and I was waiting impatiently for the PCR to make sure that I was not infected*”.

Other participants expressed that they got obsessive thoughts which led them to practice ritual behavior to get rid of those thoughts. A female physician said, “*More frankly, as a result of working with COVID-19 patients, I had a hygiene obsession, I sterilized everything around me with alcohol and avoided touching anything, I doubted the correctness of my steps while taking the PPEs off, and I was afraid of taking it off in the wrong way: for example, I suspected that I touched myself while taking it off and I felt panic, so I sterilized my whole body using alcohol*”.

• *Behavioral impact.*

Some maladaptive behaviors were adopted by our participants as a result of long working hours and stress (e.g., heavy smoking, drinking coffee). A male physician said, “*The average number of smoking cigarettes increased during the day, there was fatigue during duty… I was drinking a large amount of coffee to keep alert and maintain the quality of work*”. In addition to all of the above impacts, HCWs in the current study expressed having a learning impact; they learned many lessons from working for long hours with COVID-19 patients, such as realizing the importance of health. A female nurse said, “*life is short, everything could be changed suddenly… the most important thing in life is the person’s health… See how a small virus that cannot be seen by the eye, but did all these problems worldwide*”. Some of them learned issues related to work such as the importance of teamwork, providing support, effective communication, adaptive coping, and new experience: “*I learned the importance of teamwork to manage difficult situations and improve communications skills*” (male physician).

“*This crisis added a new experience and I consider it as a success story to tell in the future… I am proud that I was one of the first medical staff who dealt with the pandemic in its early stages*” (female nurse).

### 3.8. Theme Five: Occupational Challenges

Participants in the current study articulated many work and personal-related challenges during working with COVID-19 patients.

• *Work-related challenges.*

The patient’s response and acceptance of being diagnosed with COVID-19, dealing with patients from different age groups, and reporting PCR results to the patients were the main challenges reported by our participants. A female physician said, “*There were many challenges… All were not easy for me and for the work team as well… the acceptance of the people to be diagnosed with the disease (COVID-19) and refusing treatments were among the daily challenges*”. Reporting PCR results to patients was a great challenge health care encountered. A male physician said, “*When I wanted to inform an old man about his PCR result, I was worried and afraid of his reaction… another patient was young; however, when I informed him about the result, he got frustrated… I tried to encourage him and offer support… However, at that moment, I was stressed*”. Meanwhile, other participants communicated the effectiveness of the existing treatment as a challenge. A female nurse said, “*The biggest challenge was coming from the doubt about the efficacy of the given treatment that we provided… we had a lack of confidence in this treatment… we had a constant fear about the patient and what was the best treatment for them… at that time, the stages of disease progression were ambiguous… I think everyone had the same feeling*”.

• *Personal-related challenges.*

In addition to work-related challenges, participants were challenged by their attachment to their family and being the source of infection. A female physician said, “*My personal challenge was my attachment to my family… It was one of the biggest psychological challenges I went through, as I stopped meeting them in reality, which made me stressed, but I tried to overcome it by giving work more time… this led to results that pleased me a lot and made my family proud of me*”.

• *Social stigma related challenges.*

Most of the health care workers who engaged in the current study reported that they were challenged with facing social discrimination as a result of working with COVID-19 patients, such as blame and being avoided. A female nurse said, “*In my area, one person got the virus… Oh, that caused a complex feeling; I felt that all fingers of accusation were pointed towards me… another story happened when I went to a pharmacy to buy some stuff… the pharmacist had bothered me when he knew that I worked in the Corona department, so he showed discomfort and his facial expression meant a lot*”. A male nurse mentioned, “*People around us, when they know where I’m working… they try to avoid dealing with me… it was a difficult situation that adversely affected me*”.

Some of the participants felt social stigma, mainly when they got the infection. A female physician said, “*I felt fear and isolation after I got the Coronavirus infection… I felt that people blamed me for my infection with the disease as if I chose to be infected… They blamed me for my infection because it was my fault and my negligence of the instructions… I was very disappointed*”.

However, some of them tried to rationalize and accept the way of dealing with them in the community: “*In terms of the society view, they have the right to stay away from us. We deal with sick people continuously. The possibility of infection is high. We cannot refuse their reaction… We understand people’s concern for themselves and their families… It is a normal feeling, and it is our duty also to distance ourselves from them to reduce the spread of the virus*” (female physician).

### 3.9. Theme Six: Work-Related Concerns

• *Impact of hearing news about the collapse of health sectors in some countries.*

The collapse of health sectors in some countries and the news about it was the main concern of our participants; this news was stressful for some participants in the current study. A male physician said, “*Frankly, there was a feeling of great fear and tension that we would fall into the same thing as Italy, and as we know Jordan has limited capabilities in general but thank God… we were aware of the Ministry’s excellent work so that the Ministry of Health became called the Ministry of Corona; this matter gave us some reassurance*”. However, this news was motivating to some participants in the current study and increased their confidence in Jordan’s health care system and its ability to face the pandemic. Their confidence came from maintaining safety precautions and faith in God.

• *Governmental decision to close outpatient clinics.*

Closing the outpatient clinics was the other main concern of our participants; the HCWs in the current study agreed with the government decision in closing the outpatient clinics since it helped in controlling the spread of COVID-19 in the community; however, they reported that this decision increased their workload. A male physician said, “*It was a correct decision 100%… this prevented the spread of COVID-19 which would increase the workload pressure. Now, after opening, we received many patients and the workload increased but we managed it appropriately*”. A female physician said, “*Closing the outpatient clinics was one of the most important steps taken by the Ministry of Health. This reduced large numbers of gatherings in hospitals and relieved pressure on health care providers because the hospital is considered one of the most dangerous places at this time*”.

## 4. Discussion

This study aimed at exploring the lived experiences of physicians and nurses caring for patients with COVID-19 in Jordan given their little experience in dealing with infectious diseases. The current study provides a significant contribution to knowledge as it is one of the few studies to explore the perceptions of health care providers in Jordan towards caring for patients with COVID-19 infection. To facilitate comparing our findings with other literature, we present our discussion in a structured manner per main themes.

• **Emotional Reactions**

Jordanian HCWs feared infection and worried about their families, but they are still willing to join the battle, took up their responsibilities, concentrated on their duties, and showed professional commitment. This result is consistent with results from a Chinese study aimed at exploring the experiences of physicians and nurses towards caring for COVID-19 patients [17]. In addition to their families, HCWs were worried about spreading the infection to other patients. These worries might reflect the sense of responsibility among HCWs towards their patients. This finding is congruent with Italian and Jordanian studies which found that most of the physicians and nurses were very anxious about spreading COVID-19 to the people around [12,40]. However, the results showed that worries about dealing with COVID-19 patients diminish with time. Some of the participants started to accept the situation and work normally. This acceptance-based coping strategy was also reported in the literature [41]. The explanation behind this result might stem from the knowledge and competencies learned with time in dealing with COVID-19 patients, most importantly PPE’s.

The results showed that living with old parents is one of the factors contributing to stress among healthcare providers during the COVID-19 pandemic. This finding matches the findings of a previous study that aimed to understand nurses’ lived experiences during the COVID-19 pandemic [33]. HCWs have better levels of knowledge than the public that old people, especially those with chronic illness, are at higher risk to be infected with COVID-19.

Furthermore, inadequate training in dealing with infectious diseases was another factor that increased anxiety and stress among healthcare providers caring for patients infected with COVID-19. This finding is consistent with a study from Palestine which found that healthcare providers with no training about the response to the COVID-19 pandemic have higher levels of stress [34]. Additionally, the results showed that being away from family and friends for long hours is another factor that increases the stress among the HCWs. In fact, social support is necessary in difficult times. This result is consistent with the results of a previous study that studied the role of social support in reducing COVID-19 anxiety among nurses in the Philippines [42]. Additionally, the results showed that stress level increases while waiting for the PCR result, especially when having colleagues with positive PCR tests. This finding is judicious because the healthcare providers were in contact with two sources of infection, the infected patients, and the possible infected friends.

• **Preparation**

The results showed that the level of stress is alleviated with adequate organizational preparation in terms of PPEs’ adequacy, PCR random testing of healthcare providers, meeting the needs of healthcare providers, and providing appropriate and adequate care for colleagues who got infected with COVID-19. This finding is supported by a Canadian study which found that nurses with inadequate PPE’s are more susceptible to depression [43]. However, some healthcare providers were challenged with using PPEs for different reasons such as limitation of movement, feeling of hotness and sweating, marks on face and body after a long period of wearing PPE’s. This finding is consistent with an Indian study that studied the health and skin complications among nurses caring for COVID-19 patients [44].

In terms of personal preparation, the results of this study showed that the healthcare providers tried to increase their resilience by adhering to the necessary precautions such as handwashing, masking, and wearing gloves, in addition to some spiritual practices and positive thinking. This result was congruent with an Australian study which found that adhering to the needed precautions and doing spiritual practices are necessary to cope with the stress during the pandemic [45]. Moreover, some of the healthcare providers followed healthy lifestyles such as eating healthy food and doing physical exercises. This finding is consistent with a Turkish study which revealed that a healthy lifestyle such as quality of sleep is needed to enhance HCWs’ resilience during the COVID-19 pandemic [36]. On the other hand, some participants isolated themselves from their families. The reason behind this isolation is the fear of transmitting the virus to relatives.

• **Source of Support**

The results showed that the families, colleagues, organizations, self, and community’s support play a significant role in motivating the HCWs during the pandemic. These findings were consistent with a previous study which found that the different types of social support make the healthcare providers feel they are not alone, and more safe [17]. In addition, social support was not only necessary for motivation, but also for mental health enhancement [46]. A systematic review concluded that both personal and organizational support is accompanied by less mental distress [37].

On the other hand, our results revealed that some of the participants reported the need for another type of support, that was, the financial support. This result is congruent with a Brazilian study that concluded that nurses needed financial recognition during and after the pandemic [47]. Similarly, in a recent mixed-method study, Jordanian physicians have expressed their need for financial support/incentives while working amidst the pandemic [12]. Surprisingly, some of the healthcare providers stated that they did not need any support. This finding might be explained by the high sense of responsibility toward doing good for the community in response to the pandemic.

• **Extreme Workload**

Our study showed that long working hours is harmful to the HCWs’ social life. This result is consistent with a previous study which found that the pandemic affected the healthcare providers and made them socially isolated [48]. Our result can also be explained by the social distancing and the participants’ fear of transmitting the virus to their beloved relatives.

The long working hours with COVID-19 patients has harmed the physical health of the healthcare providers. For example, they complained of headache and muscle pain, and they started using analgesics; they complained of sleep deprivation and as a result, they used sleeping pills. Moreover, the long duty affected the HCWs’ eating habits and appetite. This finding is congruent with a review paper which concluded that healthcare providers are risky for physical complications [35]. In addition, a study from Turkey revealed that healthcare professionals may complain of sleep difficulties during the pandemic [49].

Psychologically, the long working hours have increased the healthcare providers’ anxiety, fear, and nervousness. The reasons behind the increased nervousness might be social isolation, wearing the PPEs for long hours, and the associated discomfort. Our result is consistent with a previous study which found that the pandemic has caused anxiety, depression, and suicidal ideation among the healthcare providers [48]. Additionally, a systematic review showed that HCWs are at risk to develop depressive symptoms [35].

The long working hours with COVID-19 affected the cognitive abilities of the healthcare workers, way of thinking, concentration, and thoughts. They were not able to concentrate and focus on patient care as they are supposed to. This result is in agreement with a previous study which demonstrated that problem-solving abilities are affected among the HCWs who delivered COVID-19 services [49].

In the current study, long working hours and associated stress had a behavioral impact on the HCWs. They behaved in a maladaptive way such as heavy smoking and drinking large amounts of coffee. For complicated reasons, healthcare workers usually practice at a well-known stressful environment [50,51,52], which finally impacts their well-being [53]. This result is consistent with a previous study which found that smoking is one of the maladaptive stress-coping behaviors [54].

• **Occupational Challenges**

The results showed that one of the main challenges encountered by the participants is the patient’s denial of being diagnosed with COVID-19. The results showed an additional challenge, the healthcare providers were in doubt about the effectiveness of the available treatment. The reason behind the healthcare providers’ doubt might stem from the doubts about the available treatments such as Remdesivir [55], Dexamethasone [56], and traditional Chinese medicine [57]. The HCWs in our study were also challenged by personal factors such as their attachment to family. They were worried about spreading the infection to their families, thus feeling the nightmare of being a source of infection to their families and relatives. This result is supported by previous studies that concluded the same [12,17,33,40].

Furthermore, results revealed that the participants encountered social stigma. For instance, HCWs were encountering social discrimination because of working with COVID-19 patients; sometimes they were blamed or avoided. They also felt social stigma when they got infected with COVID-19. This result is congruent with a previous study which aimed at exploring COVID-19-related stigma and its associated factors among Egyptian physicians [58].

• **Work-Related Concerns**

Our findings revealed that the HCWs were stressed because of hearing news about the collapse of health systems in some countries. This finding is congruent with a study which found that using social media as a source of information about COVID-19 is correlated with stress [59]. On the other hand, the results showed that the healthcare providers were motivated and confident in Jordan’s health care system and its ability to face the pandemic. This motivation and confidence was secondary to the strict measures implemented to fight the COVID-19 pandemic in Jordan, which proved to be effective in decreasing the infection rates [20]. Additionally, the results showed that the HCWs agreed with the government’s decision to close the outpatient clinics. Although it increased the workload, they believed that this decision helped in controlling the spread of COVID-19. Once again, a high sense of responsibility among HCWs appears in this result. This result might be explained by the worries of transmitting the virus from the hospitalized COVID-19 patients to the community through outpatient clinics.

A challenge of conducting our study was using an online platform to interview participants. It was more difficult to build a rapport with participants over the online meeting, and non-verbal cues could not be documented [60,61]. Additionally, our study reflects HCWs’ lived experiences within the Jordanian context, and this may limit the results’ transferability to other contexts. Nevertheless, considering that HCWs in different settings were exposed to a significant workload during this pandemic crisis, we expect that our findings would be helpful for policymakers and decision-makers in different countries as well. Besides, the reported experiences depend on the extent of remembering events that were faced by the HCWs; thus, recall bias could be considered.

## 5. Implications

The health sector’s policymakers are advised to consider and implement the following recommendations: (i) regulations that enhance working conditions and support occupational safety, (ii) ensuring that the mental health of the HCWs is under regular supportive monitoring by trained psychosocial staff, (iii) providing timely and accessible mental health and psychosocial support services to HCWs who are in active fight against the COVID-19, (iv) capacity building of HCWs through well-structured positive psychology training programs that enhance personal resilience and coping skills to deal with various psychosocial stressors at work (e.g., Cognitive Behavioral Therapy-based resilience training, physical activity programs, mindfulness-based interventions), (v) providing HCWs with appropriate incentives subjective to their increased workload during the pandemic crisis, (vi) continuous assessment of the health workforce needs in order to maintain sustainable working environment in healthcare settings.

## 6. Conclusions

In conclusion, HCWs in Jordan working in the wards and care centers designated for patients with COVID-19 experienced mental and emotional distress and were practicing under inadequate work conditions. This distress could be multifactorial with personal, organizational, or cultural origins. Our study findings may guide policymakers in taking into consideration the potential factors that significantly affect the working environment in healthcare settings, the physical and mental wellbeing of HCWs, and the required professional training that may help in enhancing the HCWs’ resilience and coping strategies amidst crises.

## Figures and Tables

**Table 1:** Example questions and probing that were asked during the interview, based on reviewing relevant and related literature [12,17,33,34,35,36,37].

Example Questions
Where did you work before?
For how long you have been in your current work?
Please tell me about your experiences of taking care of patients with COVID-19.
What are the differences between providing care due to the epidemic and working in your original department?
How did you feel on the first day?
Tell me about your life after you start working with COVID-19 patients.
Describe your feeling now.
What challenges did you encounter?
What external support have you received? And what other support do you need?
Can you describe the challenges you face while caring for patients with COVID-19?
Before closing the interview: Is there anything else you would like to tell me?
**Probing**
Please tell me more about that
How did you overcome these challenges?
What kind of support do you need?

**Table 2:** Demographic Characteristics of Participants (n = 26).

Variable	Hospital 2	Hospital 1	Total (%)
**Gender**			
Male	9	7	16 (61.5)
Female	5	5	10 (38.5)
**Marital status**			
Single	5	7	12 (46.2)
Married	8	6	14 (53.8)
**Job nature**			
Nurse	8	8	16 (61.5)
Physician	6	4	10 (38.5)

**Table 3:** Themes and sub-themes of lived experiences of physicians and nurses caring for COVID-19 patients in Jordan.

Theme	Sub-Themes	Classification of Subthemes (Individual vs. Organizational)
Emotional Reactions	The feeling of fears, worries, and anxiety	Individual
	Factors increased the fear, stress, and anxiety	Individual
Preparation	Organizational preparation	Organizational
	Personal preparation	Individual
Source of Support	Family and friend support	Individual
	Organizational and colleagues’ support	Organizational
	Other sources of support (community and self)	Individual
Extreme Workload	Social impact	Individual
	Physical impact	
	Psychological impact	
	Cognitive impact	
	Behavioral impact	
Occupational Challenges	Work-related challenges	Organizational
	Personal- related challenges	Individual
	Social stigma related challenges	Individual
Work-Related Concerns	Impact of hearing news about collapse of the health sector in some countries	Individual
	Governmental decision to close out-patients clinic	Organizational

## Data Availability

The dataset generated and analyzed in the qualitative interviews is not publicly available due to the potential for individual and organizational privacy to be compromised. Reasonable requests for parts of the qualitative data will be considered by the corresponding author.

## Supplementary Materials

The following are available online at https://www.mdpi.com/article/10.3390/ijerph18179002/s1. Supplementary File S1. Consolidated Criteria for Reporting Qualitative studies (COREQ): 32-item checklist (PDF. English).
